# An In Silico Omics Investigation of the Lactobacillus Genus Complex for Allergenicity Mitigation

**DOI:** 10.1002/iid3.70365

**Published:** 2026-02-23

**Authors:** Zahra Bigdeli, Ali Molaei, Nassim Rahmani, Zakie Mazhary, Najaf Allahyari Fard

**Affiliations:** ^1^ Department of Systems Biotechnology National Institute of Genetic Engineering and Biotechnology (NIGEB) Tehran Iran; ^2^ Department of Biology, Science and Research Branch Islamic Azad University Tehran Iran; ^3^ Department of Biology Shiraz University Shiraz Iran

**Keywords:** CYP450, food allergies, immunosuppressive motifs, Lactobacillus, probiotics

## Abstract

**Background:**

Among the useful methods of immune response modulation, probiotics can be included. A delicate balance in the microbiome is of extreme importance for immune homeostasis, the composition of which is influenced by lifestyle and dietary habits. The aim of this study was to identify Lactobacillus strains with potential allergenicity‐mitigating properties through comprehensive in silico analysis of their genomes and proteomes. Abrogation in such a balance may lead to the development of food allergy. So far, certain components in probiotics, including immunosuppressive motifs or metabolites like 17,18‐epoxy eicosatetraenoic acid (17,18 EpETE), seem to have the potential to reduce prevalence. However, some bacterial proteins may be allergenic or may induce cross‐reactivity.

**Methods:**

We performed an in silico study over the 161 reference genomes and proteomes of Lactobacillus. We analyzed the presence of Nucleotide immunosuppressive motifs (NISM)s, calculated the per million frequency of bases, and checked for the presence of the CYP450 enzymes/stable allergen epitopes. This was evaluated using various bioinformatics tools like Uniprot proteomes, NCBI genomes, AlgPred, PeptideCutter, EMBOSS, and MAST.

**Results:**

Our analysis identified *Latilactobacillus sakei* as a promising candidate, based on its genomic and proteomic features, for mitigating allergenicity.

**Conclusions:**

*Latilactobacillus sakei* might be a useful candidate for nutritional allergy management and related conditions. However, this potential requires verification through further experimental studies.

## Graphical abstract

This graphical abstract illustrates the most promising Lactobacillus species for reducing allergenicity through an omics approach, integrating genomics and proteomics studies. Also, provides a clear overview of candidate strains that could be effectively utilized in mitigating allergic reactions.

**Abbreviations**
DC: dendritic cell
GI: gastrointestinal
IBD: inflammatory bowel disease
IFN: interferon
MAMP: microbe‐associated molecular pattern
MPM: motif per million
NISM: nucleotide immunosuppressive motif
TLR: toll‐like receptor

## 1 Introduction

**FIGURE 1:**
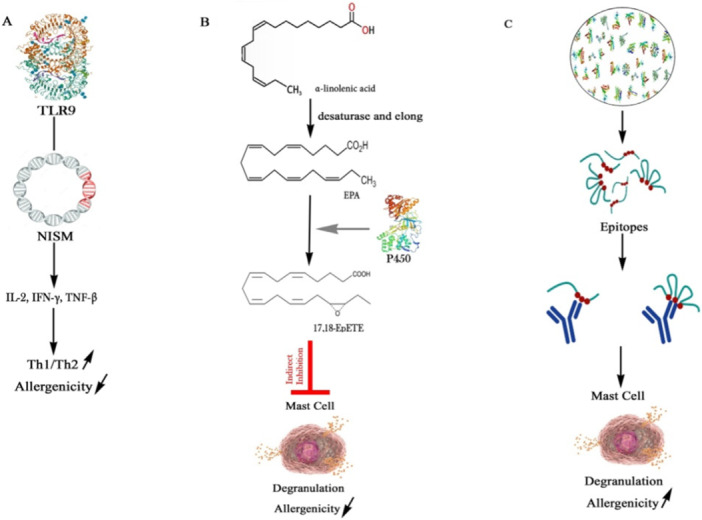
The mechanism of probiotics's effect on allergy: (A) increase of Th1/Th2 and reduction of allergenicity; (B) the effect of CYP450 on inhibition of mast cell degranulation and reduction of allergenicity; and (C) enhancement of allergenicity through epitopes.

Probiotics are live microorganisms that confer health benefits to the host [1]. Probiotics must fulfill three conditions in order to be effective: first, they must be administered in a viable form; second, the dosage must be based on clinical evidence for its efficacy; and finally, there must be a health benefit to the host [2, 3]. Moreover, the taken probiotics should be at the lowest possible taxonomic levels; if they are taken from the human gut, they have to be from normal healthy carriers to withstand the gastric enzymes and stomach acidity [4, 5, 6]. The modes of immune regulation by probiotics include the production of antimicrobial metabolites, adhesion to intestinal cells, competition for nutrients with pathogenic bacteria, reinforcement of the epithelial barrier, and acidification of the gut, which inhibits harmful microbes. The development of healthy gut microbiota is invaluable for improving the immune system [7]. Recent studies have highlighted the immunomodulatory potential of probiotics, demonstrating their ability to regulate immune responses through mechanisms such as cytokine modulation, enhancement of barrier function, and interaction with gut‐associated lymphoid tissue (GALT) [8]. About 10^14^ microorganisms, including bacteria, viruses, fungi, and protozoa, make up the human gut microbiome, which is crucial in maintaining health [9]. However, it can be disturbed by lifestyle, diet [10], synthetic chemicals, air pollutants [11], preservatives, antibiotics, and more, leading to a condition called dysbiosis, which might predispose individuals to allergic reactions [12, 13, 14]. Dysbiosis is particularly associated with the development of food allergies [15, 16]. Emerging evidence suggests that probiotics can restore microbial balance and mitigate allergic responses by promoting the growth of beneficial bacteria and suppressing pathogenic species [17]. These allergies, characterized by an immune hyper‐reactivity to specific types of food, affect approximately 10% of the world's population [18]. Their onset is influenced by genetic, environmental, and dietary factors [19, 20, 21, 22, 23, 24]. Multiple studies have identified certain strains of lactobacilli as effective probiotics for reducing food allergies [25]. For instance, specific strains have been shown to modulate dendritic cell function, enhance regulatory T‐cell populations, and reduce pro‐inflammatory cytokine production, thereby alleviating allergic symptoms [26]. A key factor in this regard is the presence of Nucleotide immunosuppressive motifs (NISM)s, specific DNA or RNA sequences that modulate immune responses. By engaging with immune cells or regulating gene expression, NISMs can prevent allergic reactions [27]. Species of Lactobacillus regulate immune responses by blocking the activation of dendritic cells and promoting the conversion of regulatory T cells, which mediate tolerance to allergens [28]. For instance, *Lacticaseibacillus casei* Shirota reduces levels of immunoglobulin E (IgE) and other food allergy markers that cause symptoms ivation. This species also suppresses T‐cell activation, helping restore balance Th1/Th2 profile in the immune system [29]. In essence, Lactobacillus species stimulate immune cells to release cytokines, such as IL‐6, TNF‐α, IL‐12, and IL‐10 [30, 31], thereby shifting the Th2 response back toward a Th1 response [32]. Additionally, Lactobacillus species are known to produce beneficial metabolites [33]. These metabolites, including short‐chain fatty acids (SCFAs) and bioactive lipids, play a critical role in immune regulation by modulating inflammatory pathways and enhancing epithelial barrier integrity [34]. Lactobacillus species produce metabolites that contain polyunsaturated fatty acids (PUFAs), which help reduce inflammation [35, 36, 37]. ω‐3 PUFAs, in particular, demonstrate anti‐inflammatory and anti‐allergic effects [38, 39, 40, 41, 42, 43, 44, 45, 46]. Omega‐3 PUFAs have both anti‐inflammatory and anti‐allergic properties [47]. These fatty acids are transformed into lipid mediators, which play critical roles in acute inflammatory responses. One such mediator, 17,18‐epoxy eicosatetraenoic acid (17,18 EpETE) [48], generated by the enzyme Cytochrome P450 (CYP450), inhibits food allergies [49, 50]. Cytochrome P450 (CYP450), found in microorganisms, plays a pivotal role in metabolizing PUFAs, leading to the production of hydroxy and epoxy fatty acids [51]. Among these, 17,18‐epoxy eicosatetraenoic acid (17,18 EpETE) has emerged as a novel lipid mediator that inhibits the progression of food allergies [52]. CYP450 converts EPA into 17,18‐EpETE, and lipid mediators produced by CYP450 have regulatory effects on inflammatory, vascular, cardiac, and renal functions [53, 54, 55]. However, care must be taken to ensure that probiotic strains do not contain allergenic proteins (epitopes). Genome and proteome analysis—the complete set of genes and proteins expressed by a bacterial strain—helps infer the bacteria's potential immune effects. Bioinformatics tools like in silico analysis enable genome and proteome minings for NISMs, CYP450, and allergenic potential. In this study, we systematically analyzed NISMs, CYP450 enzymes, and stable allergenic epitopes among Lactobacillus species. Our findings suggest that certain strains can reduce allergenicity, which could be useful in identifying potential candidates for the development of probiotic therapies for managing allergies. It is crucial to distinguish that the term “probiotic” denotes a regulated claim that requires strain‐level identification, rigorous clinical validation of efficacy, and confirmed safety in human trials, as outlined by international bodies like the EFSA and WHO/FAO [56]. The present study serves as a preliminary in silico screening of the Lactobacillus genus complex. Our objective is not to designate the strains examined herein as approved probiotics, but rather to identify species harboring genomic and proteomic features, such as NISMs, CYP450 enzymes, and a low potential for allergenicity that are associated with beneficial immunomodulatory functions. This approach aims to pinpoint promising candidates for further investigation within the stringent framework of probiotic development. The aim of this study was to identify Lactobacillus strains with potential allergenicity‐mitigating properties through comprehensive in silico analysis of their genomes and proteomes, focusing on the presence of NISMs, CYP450 enzymes, and stable allergenic epitopes (Figure 1).

## 2 Materials and Methods

### 2.1 Data Collection

We analyzed 161 reference genomes and proteomes from across the Lactobacillus genus complex in silico, using publicly available resources, such as the UniProt and NCBI databases. These databases provide extensive protein sequence and genomic data, both of which were crucial for our analysis of NISMs and allergenic potential. Curated protein sequence data is accessible through UniProt [57]. The taxonomic nomenclature for Lactobacillus species has been updated and reflects the current classification system [58, 59]. Our screening included species from Lactobacillus, Lacticaseibacillus, Limosilactobacillus, Latilactobacillus, and other related genera.

### 2.2 Identification of NISMs

A systematic approach was used to identify the presence of NISMs within the Lactobacillus genomes, based on previous studies that highlighted the role of NISMs in immune regulation. We calculated the density of NISMs per million bases across different strains, allowing us to correlate the density of NISMs with potential immunomodulatory effects. The tools EMBOSS (http://emboss.bioinformatics.nl/cgi-bin/emboss/fuzznuc) and MAST (http://meme-suite.org/doc/mast.html) were used for this analysis [60, 61]. We further examined 17 NISMs within the 161 genomes, including TCAAGCTTGA, TTAGGG, TTTCGTTT, TGGCTGTT, TTGCCGCA, GATTATCG, CGCCATTT, TTTTGCCG, TTGTCACC, CATCAAAG, AACAGCCA, TGCGGCAA, CGATAATC, AAATGGCG, CGGCAAAA, GGTGACAA, and CTTTGATG [62, 63, 64, 65]. The process involves data extraction, analysis, and comparison to determine the most prevalent NISMs. These motifs were calculated based on the Motif Per Million (MPM), which is defined as below equation:

$$\mathrm{Motif}\;\mathrm{Per}\;\mathrm{Million}\;(\mathrm{MPM})=\frac{\mathrm{Frequency}\;\mathrm{of}\;\mathrm{the}\;\mathrm{motif}\;\mathrm{in}\;\mathrm{Genome}\times \mathrm{Genome}\;\mathrm{size}\;(\mathrm{Mb})}{\mathrm{Megabases}\;\left(\mathrm{Mb}\right)(10,000,000)}$$

Genome size, measured in base pairs, varies across species due to factors like gene number, non‐coding DNA, and repetitive sequences, such as NISMs. Genome size is expressed in megabases (Mb).

### 2.3 Protein Analysis and Allergenicity Prediction

To determine the allergenic potential of proteins encoded by Lactobacillus genomes, we used AlgPred, a predictive tool for allergenicity. This algorithm integrates sequence‐based properties like hydrophobicity, charge, and structural parameters for accurate prediction of allergenic epitopes. The allergenicity and cross‐reactivity of proteins in the proteomes were assessed using AlgPred2.0, IEDB, and AllergenOnlin. AlgPred2.0 (https://webs.iiitd.edu.in/raghava/algpred/submission.html) is a computational tool designed for predicting allergenic proteins using a hybrid approach that combines machine learning algorithms with sequence‐based features. It employs several methods, including IgE epitope mapping, PID, a dipeptide composition‐based SVM module, a hybrid approach (SVMc + IgE epitope + APRs BLAST + MAST), motifs, amino acid composition, and alignment‐based methods to assess potential allergenicity. AlgPred2.0 is widely used in bioinformatics for allergenicity screening [66]. The IEDB (Immune Epitope Database and Analysis Resource) is a comprehensive resource for epitope analysis, including allergen prediction. It integrates experimental and predicted data to identify IgE‐binding epitopes, which are critical for allergenicity assessment. The database supports BLAST‐based searches against known allergenic sequences and offers tools like the Allergenicity Prediction Tool to evaluate potential cross‐reactivity [67]. AllergenOnline is a curated database and prediction tool maintained by the University of Nebraska. It uses a bioinformatics approach to evaluate protein sequences for potential allergenicity by comparing them to a reference set of known allergens. The tool follows FAO/WHO guidelines, requiring ≥ 50% identity over a stretch of 80 amino acids for positive prediction. AllergenOnline is widely used in food safety and regulatory contexts to assess the allergenic risk of novel proteins in food products [68]. These analyses helped identify allergenic epitopes, along with their accession numbers and protein names, across different Lactobacillus species. It is important to note that the allergenicity prediction tools employed in this study (AlgPred2.0, IEDB, and AllergenOnline) are primarily optimized for the identification of IgE‐binding epitopes, which are characteristic of immediate‐type allergic reactions. While this focus is highly relevant for food allergies, our in silico analysis may not capture allergenic potential mediated through non‐IgE mechanisms (e.g., T‐cell‐mediated responses).

Our in silico strategy for allergenicity prediction was designed to adhere to the weight‐of‐evidence approach recommended by international guidelines (EFSA, 2017; FAO/WHO, 2001). This involved employing multiple complementary methods: (1) a sequence‐based search for global similarity against known allergens using AllergenOnline, (2) a targeted epitope‐based analysis using AlgPred2.0 and IEDB, and (3) an assessment of protein stability under simulated gastrointestinal digestion conditions.

### 2.4 Digestion Enzyme Impact Assessment

**FIGURE 2:**
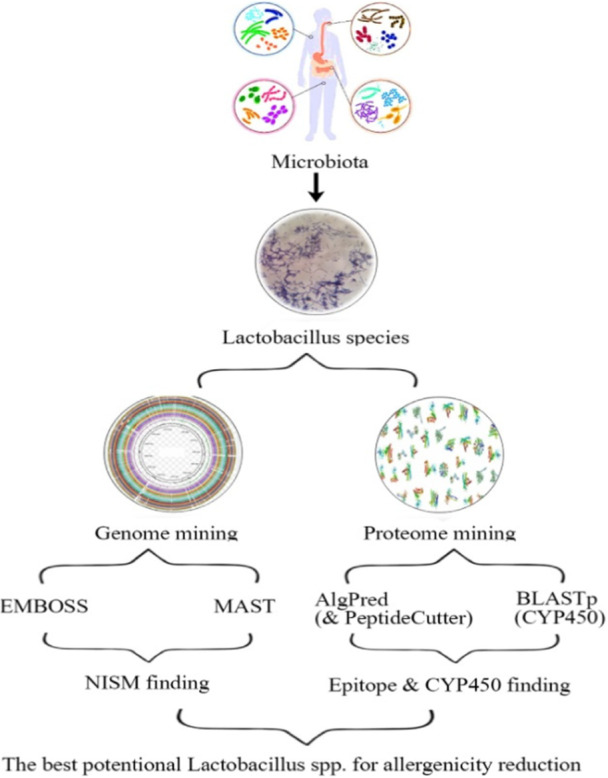
The flow chart shows the best potential Lactobacillus species for allergenicity reduction.

We assessed the stability of allergenic epitopes under digestive conditions by simulating enzymatic cleavage using PeptideCutter (https://web.expasy.org/peptide_cutter) [69]. The effects of pepsin, chymotrypsin, and trypsin—enzymes found in the gastrointestinal tract—were analyzed to evaluate how digestive processes might reduce the allergenicity of probiotic proteins. Stable epitopes that remain after digestion could potentially cause allergies. PeptideCutter was used to identify cleavage site positions and the number of cleavages, assessing enzyme efficacy. Figure 2 provides a summary of the bioinformatics workflow [70].

## 3 Results

### 3.1 The 10 Highest‐Ranking NISMs

**FIGURE 3:**
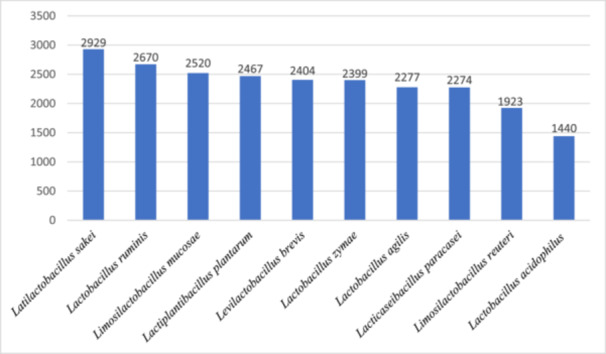
The total MPM of NISMs in the top 10 Lactobacillus species.

Among the Lactobacillus species analyzed, it is observed that the top 10 species with the highest MPM for NISMs include *Latilactobacillus sakei, Lactobacillus ruminis, Limosilactobacillus mucosae, Lactiplantibacillus plantarum, Levilactobacillus brevis, Lactobacillus zymae, Lactobacillus agilis, Lacticaseibacillus paracasei*, and *Limosilactobacillus reuteri* (Figure 3).

### 3.2 Bacterial Strain Selection and Data Retrieval

Initial screening of the UniProt database identified approximately 1900 entries classified under the Lactobacillus genus complex. From this extensive list, we refined our selection to include 161 well‐characterized species and strains for which high‐quality reference proteomes were available in the Reference Proteomes section of UniProt (www.uniprot.org) [71]. The taxonomic identity of each selected species was further verified and authenticated using the NCBI Protein database (www.ncbi.nlm.nih.gov/protein) to ensure nomenclature accuracy consistent with the latest taxonomic revisions.

### 3.3 Identification of Allergen and Stable Epitopes

We used AlgPred2.0, IEDB, and AllergenOnline to predict the allergen epitopes of each of the selected Lactobacillus species. The stable allergen epitopes were also predicted using PeptideCutter [72]. In Table 1, an overview is given of the number of allergens and stable allergen epitopes found in the top 10 Lactobacillus species.

**TABLE 1:** The number of identified allergen epitopes and stable allergen epitopes obtained from UniProt, AlgPred2.0, IEDB, AllergenOnline, and PeptideCutter.

Species	Proteins	Allergen epitopes	Stable allergen epitope
*Latilactobacillus sakei* subsp. sakei (23 K)	1872	21	0
*Lactobacillus ruminis* (ATCC 27782 RF3)	1851	20	0
*Limosilactobacillus mucosae* LM1	1970	27	0
*Lactiplantibacillus plantarum* (ATCC BAA‐793 NCIMB 8826 WCFS1)	3087	44	0
*Levilactobacillus brevis* (ATCC 367 JCM 1170)	2201	29	2
*Lactobacillus zymae* DSM 19395	2385	28	0
*Lacticaseibacillus paracasei* (ATCC 334 BCRC 17002 CIP 107868 KCTC 3260 NRRL B‐441)	2708	33	2
*Lactobacillus agilis* (DSM 20509)	1951	28	0
*Limosilactobacillus reuteri* (DSM 20016)	1865	20	1
*Lactobacillus acidophilus* (ATCC 700396 NCK56 N2 NCFM)	1859	10	0

*Lactiplantibacillus plantarum, Lacticaseibacillus paracasei, Levilactobacillus brevis, Lactobacillus agilis* DSM 20509, and *Lactobacillus zymae* DSM 19395 have 44, 33, 29, 28, and 28 allergen epitopes, respectively. On the other hand, *Lactobacillus acidophilus*, has the lowest number of allergen epitopes, with 10 allergen epitopes. After using PeptideCutter, it was found that *Lacticaseibacillus paracasei, Levilactobacillus brevis*, and *Limosilactobacillus reuteri* have the highest number of stable allergen epitopes, with 2, 2, and 1 stable allergen epitopes, respectively. No stable allergen epitopes were found in other Lactobacillus species (Tables 2 and 3).

**TABLE 2:** IgE epitope of *Lactobacillus acidophilus* proteins (strain ATCC 700396) using AlgPred.

No	IgE epitope	Sequence matched	Position	Number of repetitions
1	ALEAYA	ALEAYG	1080	1
2	GVQEGAKALY	LVQEGAIQLY	418	1
3	IDRSLPPIVK	KDGSLPPIIK	374	1
4	NGELIRRAKSAEK	NDDLIRRRKDAQK	47	1
5	GVQEGAKALY	GVADGAKALK	190	1
6	IDQIEKQAKD	TDQIEKFAKH	523	1
7	TPGQFEDFFP	TPGRFFDFFS	120	1
8	NNFGKLFEVK	NNDGISFEVK	504	1
9	AEEVEEERLK	AEEVEEIRQF	45	1
10	ALEAYA	ALEAYQ	165	1

**TABLE 3:** Accession number, protein name, and protein digestion analysis using PeptideCutter.

Accession number	Protein name	Check digestion protein
Q5FM97	DNA‐directed RNA polymerase subunit beta	Yes (1080–1085)
Q5FLA9	Peptide chain release factor 3	Yes (418–427)
Q5FHT6	Putative lipoprotein A‐antigen	Yes (190–199)
Q5FM07	DNA polymerase III subunit gamma/tau	Yes (523–532)
Q5FLC8	ATP‐dependent RNA helicase, DEAD‐DEAH box	Yes (120–129)
Q5FKC2	Excinuclease subunit A	Yes (504–513)
Q5FLB2	Regulatory protein RecX	Yes (45–54)
Q5FMY0	Uncharacterized protein	Yes (165–170)

### 3.4 Detection of Cytochrome P450

However, putative Cytochrome P450 genes were detected in the genomes of *Secundilactobacillus acidipiscis* and *Latilactobacillus sakei*, but were absent in the other species within the Lactobacillus genus complex analyzed in this study.

## 4 Discussion

Allergens originate from diverse sources, including mites, pollen, molds, animal substances, foods, venom, and latex. All of these allergens can stimulate the immune system, which includes high‐affinity IgE antibodies and results in allergic reactions among others [73]. Cross‐reactivity occurs through recognizing related proteins from a source with at least 70% sequence similarity when using IgE antibodies directed against one allergen [74, 75]. Food allergies, in particular, can cause both mild discomfort and severe, life‐threatening reactions. Th2‐type immune response has been linked to the development of food allergies, in which a misbalance of the T helper cell type 1 and 2 (Th1 and Th2) pathways results in these allergic conditions [75, 76]. This imbalance may be corrected by NISMs, abundant in the genomes of Lactobacillus species [77]. The 10 Lactobacillus species with the highest NISM densities, as determined by MPM calculations, are presented in Figure 3. More specifically, it has been shown that ω‐3 PUFAs have immunosuppressive effects on cell‐mediated immune responses [78]. ω3 PUFA is metabolized to docosahexaenoic acid (DHA) and eicosapentaenoic acid (EPA), which get converted to pro‐resolving anti‐inflammatory lipids. A novel lipid mediator, 17,18‐epoxy eicosatetraenoic acid (17,18 EpETE), was found to inhibit food allergy development [79, 80, 81]. CYP‐derived lipid mediators are crucial in mediating the cross‐talk between inflammatory responses, as well as cardiac, vascular, and renal functions. 17,18‐EpETE is synthesized from EPA by CYP [39, 82, 83]. So, we wanted to establish the presence of this enzyme because its role in synthesizing 17,18‐EpETE reduces the allergenic property in probiotics. In so doing, it was established that *Latilactobacillus sakei* and *Secundilactobacillus acidipiscis* have Cytochrome P450, which is one of the probable factors.

Yi‐Fan Hong et al. aimed to study the immune‐regulatory ability of *Lactiplantibacillus plantarum* and *Latilactobacillus sakei* both isolated from kimchi. Their results suggested a crucial role for *Latilactobacillus sakei* in modulating the inflammatory response, which could provide some protective effects against over‐inflammation [84]. Snel et al. tested the strain‐specific immunomodulation of *Lactiplantibacillus plantarum* on birch pollen‐allergic individuals out of season. They showed that not all *Lactiplantibacillus plantarum* exerted the same degree of immunomodulating activity at the level of immune responses in allergic individuals. Their results indicated that some strains could enhance the response of regulatory T cells and modulate cytokine production, thus enabling a decrease in allergic symptoms elicited by exposure to allergens. This emphasizes the importance of selecting proper probiotic strains for therapeutic action in allergic conditions [85]. Dong et al. carried out a study to evaluate the immunomodulatory effects of a probiotic drink containing *Lacticaseibacillus casei* Shirota (LcS) in enhancing immune functions in healthy older adults. The study also identified a higher trend ratio between the anti‐inflammatory cytokine IL‐10 and the pro‐inflammatory cytokine IL‐12 after LcS intake. The result favors promoting an increased anti‐inflammatory state, which may be health‐promoting and involved in combating inflammatory conditions attributed to the aging process. The findings reemphasize the immunomodulatory potential of probiotics, particularly *Lacticaseibacillus casei*, as part of dietary intervention aimed at increasing immune function in older adults. More importantly, with the increasing aging population and challenges of immunosenescence, the increased incorporation of probiotics into the diet may benefit health outcomes in this sector [86].

Our findings not only highlight the diversity of immunomodulatory potential within the Lactobacillus genus complex but also provide a foundational framework for screening this broad group of lactobacilli for safety and efficacy. While the presence of beneficial features like NISMs and cytochrome P450 enzymes helps identify promising candidates, our analysis reveals that this alone is insufficient for a comprehensive safety assessment. Crucially, the safest species should ideally lack stable allergen epitopes. Through in‐depth sequence analysis of 53,438 proteins, we identified 557 allergen epitopes, of which only 17 remained stable after simulated enzymatic digestion. In silico digestion analysis using PeptideCutter demonstrated that most of the predicted allergen epitopes were susceptible to enzymatic cleavage by pepsin, chymotrypsin, and trypsin. Furthermore, we observed that some species, notably *Latilactobacillus sakei*, possessed no stable allergen epitopes, underscoring their potential as particularly safe candidates for future development.

Furthermore, it is imperative to acknowledge the limitations inherent in our in silico approach. Our allergenicity prediction primarily focused on IgE‐mediated mechanisms using tools like AlgPred2.0 and AllergenOnline. However, allergic responses can be mediated through non‐IgE pathways (e.g., T‐cell‐mediated reactions), and the potential of peptides to influence MHC‐mediated cytokine polarization (e.g., toward pro‐allergenic IL‐4 or regulatory IL‐10) remains unexplored here. Future studies should incorporate advanced immunoinformatic tools to predict T‐cell epitopes and cytokine profiles for a more comprehensive risk assessment.

Moreover, the immunomodulatory potential of a probiotic strain is a complex balance influenced by factors beyond the scope of this genomic screen. While we identified beneficial features like NISMs and putative CYP450 enzymes, the overall functional outcome in the host depends on the net effect of various metabolites, including immunomodulatory peptides, polysaccharides, and SCFAs, which may exert stronger protective effects than the potential risk posed by limited IgE‐reactive epitopes. This balance is highly strain‐specific and dynamically regulated by environmental conditions in the gut (e.g., low pH, bile salts, and digestive enzymes). These stresses can induce the expression of moonlighting proteins, whose altered conformation and function could potentially unveil or mask allergenic epitopes not predictable by sequence analysis alone. Therefore, the absence of predicted IgE epitopes does not unequivocally guarantee non‐allergenicity.

Finally, it is crucial to note that the presence of a CYP450 gene, as identified in *Latilactobacillus sakei*, indicates potential but not proven function. Its expression and ability to metabolize dietary PUFAs into anti‐inflammatory mediators like 17,18‐EpETE are contingent upon environmental factors and substrate availability. Thus, our findings necessitate rigorous functional validation through in vitro assays using stress‐exposed bacterial cells, transcriptomic/proteomic analyses to confirm gene expression, and in vivo models to evaluate the overall immunomodulatory profile and true safety of these promising candidates under physiologically relevant conditions.

While this study provides computational insights into the allergenic potential and immunomodulatory mechanisms of Lactobacillus strains, an important limitation is the lack of experimental validation of the in silico predictions. Further experimental studies are needed to validate the predicted epitopes and clarify the functional contributions of key enzymes like cytochrome P450.

## 5 Conclusion

In conclusion, our in silico screening identifies *Latilactobacillus sakei* as a promising candidate among the expanded Lactobacillus genus complex for further investigation, due to its high MPM for NISMs, putative cytochrome P450, and lack of stable allergen epitopes. These genomic features suggest it might pose a low risk of IgE‐mediated sensitization and could potentially contribute to allergenicity reduction through the production of anti‐inflammatory metabolites. Indeed, earlier studies have supported that intake of *Latilactobacillus sakei* has been positively associated with clinical improvement in children diagnosed with atopic eczema–dermatitis syndrome. However, it is crucial to emphasize that this potential must be verified through functional assays that evaluate the overall balance of its immunomodulatory outputs, including the production of protective metabolites like SCFAs and anti‐inflammatory peptides, under physiologically relevant conditions.

Therefore, while this research puts forth *Latilactobacillus sakei* as a very promising candidate for future study, more experimental research is necessary to fully understand its therapeutic potential and safety profile for allergic diseases. The suggested directions include: (1) Search for biomarkers, (2) Mechanistic studies of metabolites, including 17,18‐EpETE, (3) Extended comparative genomic analysis, (4) Cross‐reactivity studies, and (5) Clinical trials. These studies will be essential to comprehensively evaluate its role in allergy management and to firmly establish it as a reliable candidate for safe probiotic supplements.

## Author Contributions

**Zahra Bigdeli:** data curation, investigation, software. **Ali Molaei:** formal analysis, writing – original draft. **Nassim Rahmani:** formal analysis, investigation. **Zakie Mazhary:** data curation, investigation. **Najaf Allahyari Fard:** conceptualization, project administration, validation, writing – review and editing.

## Ethics Statement

The authors have nothing to report.

## Consent

The authors have nothing to report.

## Conflicts of Interest

The authors declare no conflicts of interest.

## Data Availability

The datasets generated and/or analyzed during the current study are not publicly available due to their inclusion in ongoing research projects but are available from the corresponding author upon reasonable request.
